# Editorial: Investigating the role of biological pathways involved in brain disorder and infection

**DOI:** 10.3389/fphar.2023.1217333

**Published:** 2023-05-24

**Authors:** Ramanathan Srinivasan, Xiangmin Lin, Natarajan Suganthy, Balakrishnan Shanmuganathan, Kundu Somanath

**Affiliations:** ^1^ Centre for Research, Centre for Materials Engineering and Regenerative Medicine, Bharath Institute of Higher Education and Research, Chennai, Tamil Nadu, India; ^2^ School of Life Sciences, Fujian Agriculture and Forestry University, Fuzhou, China; ^3^ Bionanomaterials Research Lab, Department of Nanoscience and Technology, Alagappa University, Karaikudi, Tamil Nadu, India; ^4^ Department of Brain Sciences, Faculty of Medicine, Imperial College London, London, United Kingdom; ^5^ Department of Neuroscience and Physiology, SUNY Upstate Medical University, Syracuse, NY, United States

**Keywords:** aging, infectious disease, neurodegeneration, vascular dementia, stroke, epilepsy, neuroinflammation

## Background

Understanding the mechanism behind brain disorders is still challenging. The phrase “brain disease” encompasses a range of neurological illnesses that are a significant cause of disability and the second leading cause of mortality in the modern world. Despite the protective measures such as the blood-brain barrier, the skull, the membrane meninges, and the cerebrospinal fluid, bacterial infections can still occur, leading to conditions like meningitis, which involves inflammation of the brain or spinal cord lining. Aging plays a crucial role in the development of brain disorders. It contributes to blood vessel structural changes, affecting blood flow to peripheral organs and leading to the breakdown of the blood-brain barrier. These changes ultimately result in cognitive and sensorimotor impairment, leading to conditions like vascular dementia. Adult brain tumors are also very commonly seen with aging.

Unfortunately, despite advancements in clinical and preclinical research, there are currently no viable treatment approaches to manage or prevent brain-related illnesses. The Research Topic “Investigating the role of biological pathways involved in brain disorder and infection” aims to address this gap. It consists of 6 articles, including 5 original research and 1 review articles, authored by more than 49 researchers. The objective of this Research Topic is to compile a Research Topic of articles focused on the pathways and mechanisms underlying neurological diseases. Additionally, it aims to identify viable biomarkers and explore cutting-edge treatment approaches for these brain illnesses.

One crucial aspect of brain physiology involves gangliosides, which are essential components of the plasma membrane. These gangliosides play a pivotal role in promoting various physiological processes within the brain (Magistretti et al., 2019). Studies have reported alterations in the relative abundance of specific gangliosides in neurodegenerative diseases (Blennow et al., 1992). However, the cellular and molecular processes underlying ganglioside GM1 in the brain are not yet well understood. To shed light on this topic, Finsterwald et al. conducted a study examining the impact of ganglioside GM1 on the metabolism of astrocytes and neurons. They utilized co-cultures as well as monocultures of each cell type to investigate the effects. The researchers discovered that ganglioside GM1 controls astrocyte glucose metabolism, leading to glucose absorption, glycogen mobilization, lactate secretion, and regulation of genes involved in glucose metabolism. Additionally, they found that ganglioside GM1 activates neuroprotection genes and enhances mitochondrial activity in neurons. Importantly, these effects were dependent on the presence of astrocytes.

Mitochondria are crucial for energy production, and incorporating bacteria-derived mitochondria enhances efficiency by connecting cytochrome c oxidase and F-ATPase (Osellame et al., 2012). However, this process also generates reactive oxygen species (ROS), which can have both positive and negative effects on the host. Recent studies reveal a pro-inflammatory aspect in neuropsychiatric disorders, where ROS can cause damage and cognitive dysfunction (Simpson and Oliver, 2020). Cognition relies on an energy-consuming neuronal network, so any disruptions in mitochondrial function can impact brain energy supply, metabolite generation, and cognitive processes. Mitochondrial abnormalities and oxidative stress are linked to psychiatric disorders like schizophrenia and bipolar disorder (Morella et al., 2022). Büttiker et al. have highlighted the role of energy in cognition, indicating that mitochondrial function plays a crucial part in the processes that contribute to maladaptive cognitive functioning and psychiatric symptoms. These processes involve the movement of energy within the brain and the build-up of oxidative stress byproducts. Additionally, they propose a hypothesis that neuropsychiatric symptoms could be manifestations of ancient evolutionary responses employed by both hosts and pathogens, aimed at self-repair and proliferation through parasitic or symbiotic mechanisms.

Prevalent deadly symptoms in intensive care units, sepsis, and septic shock, have dreadful consequences, particularly sepsis-associated encephalopathy (SAE), affecting a significant percentage of sepsis patients (7%–71%) and substantially increasing fatality rates (Gofton and Young, 2012). The defining feature of SAE is brain damage, which can have long-term impacts on cognitive and psychological processes (Iwashyna et al., 2010). Despite extensive research on diagnostics, clinical development, and pathological mechanisms, critical gaps persist in our understanding of the underlying mechanisms, early brain damage, and prompt management for sepsis patients. To gain insights into the biological processes and molecular causes of cognitive and neurofunctional impairments, researchers have employed various omics methods such as expression arrays, transcriptomics, and metabolomics. In this Research Topic, Xu et al. conducted an RNA-seq and metabolomics-based integrated analyses to explore potential pathophysiological pathways in the hippocampus by administering LPS to mice via intraperitoneal injection. This comprehensive investigation revealed significant changes in inflammatory, neuroinflammatory, synaptic, and metabolic pathways in the hippocampal tissues exposed to LPS.

Women are more susceptible to Alzheimer’s disease (AD) compared to men of the same age, with the postmenopausal stage and declining estrogen levels being linked to this increased risk. Progesterone deficiency has been associated with the development of amyloid plaques, activation of the NF-kB pathway, and neuroinflammation, contributing to cognitive impairment (Hamson et al., 2016). Furthermore, a high-fat diet (HFD) has been shown to elevate the risk of certain diseases and worsen cognitive impairment by damaging mitochondria and synapses in estrogen-deficient mice (Pratchayasakul et al., 2015). The pathogenesis of AD involves the gut microbiota, which acts as a dietary sensor and influences cognitive impairment. Extensive research has focused on epigallocatechin-3-gallate (EGCG), the primary catechin in green tea. Previous studies demonstrated that EGCG reduces cognitive impairment in estrogen-deficient mice at 5 months of age (Zhang et al., 2021). However, it remained unclear whether EGCG could alleviate cognitive impairment in mice fed with HFD without estrogen. Qu et al. made an important finding that demonstrated the beneficial effects of EGCG, which effectively slowed cognitive deterioration in ovariectomized (OVX) mice fed with HFD. They also observed that EGCG prevented the decrease in relative abundance of *Prevotella* (in Bacteroidetes) and the increase in relative abundance of *Bifidobacteria* (in Actinobacteria). The cognitive enhancement effect of EGCG on HFD-fed OVX mice was mediated by five functional gut microbiota genes. These findings highlighted the potential of EGCG to mitigate cognitive impairment in mice fed with HFD without estrogen and emphasize the role of the gut microbiota in mediating the cognitive benefits of EGCG.

In recent decades, several respiratory viruses, including the influenza A virus (IAV), have posed significant threats to global health and economies. IAV is a major respiratory tract infection that leads to hospitalizations and fatalities worldwide. It has been associated with neurological disorders and can cause severe brain inflammation, encephalitis, in certain neurotropic strains such as H7N9, H7N7, and H5N1 (Ludlow et al., 2016). These neurotropic IAVs can invade the central nervous system, leading to neuroinflammation driven by microglia. Preventative and treatment measures are crucial to address these issues. A study made by Demuth et al. comparing young and old mice explored the protective benefits of vaccination against acute neuroinflammation and subsequent neuronal damage caused by H7N7 infection, particularly in the sensitive hippocampus region of the brain. The findings revealed that while the impact of vaccination was greater in young mice due to immunosenescence and inflammaging in older animals, previously immunized mice showed improved survival and reversal of specific synaptic loss caused by the infection. Despite initial disease symptoms and higher neuroinflammatory aspects in older mice, vaccination proved effective in combating the effects of the virus. Considering the vital role of the nervous system in regulating essential bodily functions, vaccination is recommended as an intervention to mitigate the effects of the virus.

Ferroptosis, a regulated form of cell death characterized by lipid peroxidation and iron accumulation, has garnered significant interest in academic research. Previous studies have focused on ferroptosis without specific classification in the field of cancer and stroke research (Zhao et al., 2022). However, the role of ferroptosis in brain science remained unclear. To address this, a bibliometric study made by Miao et al. analyzed the development of ferroptosis research in the brain. Over the period from 2012 to 2021, 655 papers on ferroptosis in the brain were published, showing a continuous growth in recent years. Prominent contributions came from the Universities of Melbourne and Pittsburgh, with the majority of articles originating from China and the United States. Keyword analysis revealed the frequent use of terms such as “amyloid precursor protein,” indicating its significance in the field.

The studies compiled in this Research Topic contribute to the expanding body of knowledge in understanding brain disorders and infections. By shedding light on the intricate mechanisms involved, they provide invaluable insights into the pathophysiology of these conditions. Moreover, they offer potential directions for future research and therapeutic interventions in the field of neuroscience.
